# Risk Assessment of Florists Exposed to Pesticide Residues through Handling of Flowers and Preparing Bouquets

**DOI:** 10.3390/ijerph14050526

**Published:** 2017-05-13

**Authors:** Khaoula Toumi, Laure Joly, Christiane Vleminckx, Bruno Schiffers

**Affiliations:** 1 Gembloux Agro-Bio Tech/ULg—Pesticide Science Laboratory, Passage des Déportés 2, 5030 Gembloux, Belgium; bruno.schiffers@ulg.ac.be; 2 Operational Direction Food, Medecines and Consumer Safety, Institut Scientifique de Santé Publique, Rue Juliette Wytsman 14, 1050 Brussels, Belgium; Laure.Joly@wiv-isp.be (L.J.); Christiane.Vleminckx@wiv-isp.be (C.V.)

**Keywords:** pesticide residues, dermal exposure, risk assessment, cut flowers, florists

## Abstract

Flowers are frequently treated with pesticides and, as a result, florists handling daily a large number of flowers can be exposed to pesticide residues. A study was conducted among twenty volunteer florists located in Namur Province and in the Brussels Capital Region of Belgium in order to assess their potential dermal exposure to dislodgeable pesticide residues transferred from flowers to hands. Two pairs of cotton gloves were worn during two consecutive half days while handling flowers and preparing bouquets (from min 2 h to max 3 h/day). The residual pesticide deposits on the glove samples were extracted with a multi-residue Quick Easy Cheap Effective Rugged Safe (QuEChERS) method and analyzed by a combination of gas and liquid chromatography tandem mass spectrometry (GC-MS/MS and LC-MS/MS) by an accredited laboratory. A total of 111 active substances (mainly insecticides and fungicides) were detected, with an average of 37 active substances per sample and a total concentration per glove sample of 22.22 mg/kg. Several predictive levels of contamination were considered to assess the risk. The potential dermal exposures (PDE) of florists were estimated at the average, for different percentiles, and at the maximum concentration of residues in samples. At the PDE P90 and at the PDE_MAX_ (or worst case) values, three and five active substances respectively exceed the Acceptable Operator Exposure Level (AOEL), indicating risk situations. For the systemic exposure (SE), one active substance (clofentezine) exceeds the AOEL at the P90 predictive level. In the worst case, SE_MAX_ (at the maximum concentrations), four active substances (clofentezine, famoxadone, methiocarb, and pyridaben) exceed their respective AOEL values. Among the 14 most frequently detected active substances, two have SE_MAX_ values exceeding the AOEL. Exposure could be particularly critical for clofentezine with an SE_MAX_ value four times higher than the AOEL (393%). The exposure of florists appeared to be an example of a unique professional situation in which workers are exposed regularly to both a very high number of toxic chemicals and rather high concentration levels. Therefore the priority should be to raise the level of awareness among the florists who must change their habits and practices if they want to minimize their exposure.

## 1. Introduction

Flower production generally involves frequent use of a wide range of pesticides to control diseases and pests in an effort to reduce or eliminate yield losses and maintain high product quality [1,2]. A great majority of producers consider the use of pesticides as necessary to achieve their production targets and the only way to be able to market large quantities of floral products with an acceptable quality and relatively modest price. Research published in 1979 showed that 18 of 105 lots (17.7%) of all flowers imported into Miami contained pesticide residue levels greater than 5 ppm and that three lots had levels greater than 400 ppm [3]. Thirty-seven years later, recent studies on cut flowers (roses, gerberas, and chrysanthemums) sold in Belgium showed that flowers are heavily contaminated. One hundred and seven active substances (a.s.) were detected, i.e., an average of almost 10 active substances per sample and a total average pesticide load of 15.72 mg/kg of flowers [4]. In another study, a total of 97 actives substances were detected on 50 bouquets of roses [5].

On one hand, weakness of local regulations and the lack of maximum residue limits (MRL) for flowers explains that, unlike other crops which are harvested for consumption, there is less restriction on the use of pesticides on flowers. Cut flowers tend to be sprayed at the maximum allowed dosage up to the time of harvest, often with rather toxic chemicals, and then shipped directly to the markets with no interval between treatment and harvest. According to Rikken [6], a vast majority of the producers and European florists are not actively engaged in social and environmental standards, either when they purchase flowers or in communication with their clients. When selling products via the auction system, there are no mandatory requirements with respect to certifications such as the MPS-ABC (Milieu Project Sierteelt A, B and C) Standard, FFP (Fair Flowers-Fair Plants), or Florimark TraceCert [6].

Despite their popularity and extensive use, pesticides may present serious health concerns for exposed operators and workers. Many research studies have demonstrated both acute and chronic toxic effects after exposure during spraying or in post-harvest operations. Moreover, a recent study has shown that pesticides may also have negative impacts on the public health in general [7]. The United Nations Environment Program (UNEP) estimates that, in the whole world, approximately 20,000 workers die every year from pesticide poisoning after direct or indirect exposure [8,9]. No one can deny today that pesticides can be an important source of injury and illness among workers and other professionals who are not informed about the toxicity of the plant protection products, not properly protected, and exposed regularly to pesticides during their usual activities.

Over the past decade, several studies have pointed to exposure to pesticides as a potential cause of prostate and testicular cancers among male floriculturist pesticide applicators and of cervical cancer among females in Florida [10]. In Italy, early-stage cancers have been observed in 60 percent of long-term workers in the floriculture industry [11]. Around the world, genetic damage has been reported in more than 71 percent of cut flower growers [12]. The prevalence of reproductive problems (abortion, prematurity, and congenital malformations) has been reported in Colombian workers who had been working in the floriculture industry for at least six months [13]. In the Philippines, a study assessed the risk factors to pesticide exposure and reported that the most commonly associated health symptoms among cut-flower farmers are weakness and fatigue, muscle pain, chills and fever, blurred vision, dizziness, and headache [14].

The exposure of workers must be estimated for activities that involve contact with treated crops or products (e.g., picking, harvesting, cutting, maintenance, inspection, irrigation). Worker exposure can arise from other activities such as packaging, sorting, and bundling [15]. Considering the numerous and high levels of pesticide residues found on cut flowers in our previous study [4], the florists could be a group more severely exposed to serious hazards than other workers. This is a unique risk situation where workers could be exposed almost every day to many different pesticide residues during their professional activities. This is the reason why a risk assessment was deemed necessary to evaluate the health risks of people who manipulate contaminated flowers. Based on the results, it will be considered if recommendations to reduce the exposure by appropriate prevention and protection measures need to be established.

The amount of pesticide residues on the hands of workers represents the main measure of dermal exposure [16]. As skin is generally recognized as the primary route of exposure to pesticides [17,18,19,20,21,22], the transfer of pesticide residues to the hands could contribute significantly to the total exposure. According to their physical and chemical properties (physical state, vapor pressure, Henry constant, solubility, hydrolysis rate), many pesticides sprayed on cut flowers are in the form of persistent, fat-soluble pesticide residue, which can be dislodged from the two-sided foliar surface of a plant or after spraying. The active substances are adsorbed and fixed on the surface of the plant and therefore could be dislodged by contact with hands. The actual dermal exposure has been defined previously as the amount of pesticide coming into contact with the skin of workers that becomes available for absorption through the skin [23,24,25]. Recently, the EFSA (European Food Safety Authority) [15] has harmonised the approach of pesticide exposure assessment for workers. The EFSA has proposed various transfer coefficients [26,27] to be applied for different scenarios (nature and duration of the activity during re-entry), including activities in ornamentals [15]. In this paper, we have attempted to measure the transfer of pesticide residues from flowers to hands and to assess the potential dermal exposure of florists. Finally, the risk level for each active substance was established by comparison to the Acceptable Operator Exposure Level (AOEL) [28].

## 2. Materials and Methods

### 2.1. Assessment of Florist Hands Exposure Using Cotton Gloves

Cotton gloves can be used to assess the potential dermal exposure of workers through hands. Gloves worn during normal professional tasks act as a reservoir for active substances that come into contact with the skin [18,29,30]. A study was conducted among twenty volunteer florists located in Namur Province and in the Brussels Capital Region of Belgium to evaluate their potential dermal exposure, measuring the potential transfer of pesticides from treated flowers to hands. Two pairs of 100% cotton gloves were distributed to each florist and worn during two consecutive half days when handling flowers and preparing bouquets (from min 2 h to max 3 h/day). The two pairs were collected as a single sample (four gloves/sample), weighed, cut in small pieces with scissors, and stored in freezing bags at −18 °C until transport (by road, from Gembloux to Ghent) and analysis.

### 2.2. Extraction and Analysis of Pesticide Residues

The residual pesticide deposits on the gloves were analysed by PRIMORIS (formerly FYTOLAB, Technologiepark 2/3, 9052 Zwijnaarde, Belgium) laboratory holding a BELAC (Belgian Accreditation Council) accreditation to ISO/CEI 17025 for pesticide residues. PRIMORIS is an independent, accredited, and officially recognized service laboratory (accreditation number 057-TEST). Samples were analyzed using a multi-residue Quick Easy Cheap Effective Rugged Safe (QuEChERS) method validated by the laboratory for the analysis of residues in foodstuffs. It detects approximately 500 different active substances (a.s.) in a single analysis thanks to a combination of gas and liquid chromatography. The extraction procedure is based on the AOAC (Association of Official Analytical Chemists) Official Method 2007.01. [31]. Briefly, a homogenous 10.0 g sub-sample (small pieces of gloves) is weighted into a 50 mL polypropylene tube. Then, 10 mL of acidified acetonitrile (1% acetic acid), 4 g of anhydrous magnesium sulfate (MgSO_4_), and 1 g of sodium acetate (NaOAc) are added. After shaking and sonication in a ultrasonic bath, the polypropylene tube is centrifuged. A portion of the acetonitrile phase (upper layer) is transferred to vials and further analyzed by gas or liquid chromatography tandem mass spectrometry (GC-MS/MS (Thermo Fischer Scientific, Interscience Belgium, Louvain-la-Neuve, Belgium) or LC-MS/MS (Waters Corporation, Zellik, Belgium)), according to the active substances to be determined (GC-MS/MS for small, thermally stable, volatile, non-polar molecules, or LC-MS/MS for larger, thermolabile, non-volatile, or polar molecules). For almost all active substances, the limit of quantification (LOQ) was ≤0.01 mg/kg. A similar method has been previously used to measure the pesticide deposits on flowers [4]. Considering that extraction from flowers could differ from the one from cotton gloves, a preliminary multi-residue recovery study has been carried out. Therefore the analytical results were corrected accordingly for all active substances with a recovery ratio between 50–130% (only few substances had a percentage of recovery below or above these values; in this case, the results remain uncorrected in the tables).

### 2.3. Multi-Residue Recovery Preliminary Study

To assess the recovery percentages of pesticide residues, two multi-pesticide solutions were spiked on cotton gloves, which were allowed to dry for 24 h, cut in small pieces, stored, and analyzed with a similar multi-residue method. The recovery is obtained as a ratio between the amount of residue measured in the extract after analysis and the amount spiked on gloves (% Recovery = (amount of extracted residues (mg)/amount of active substance placed on the gloves (mg)) × 100). Three replicates of the recovery trial were conducted on three different days. For each trial, four samples were prepared (5 g of gloves/sample); one sample with pieces of untreated gloves (blank sample) and three samples used to estimate recoveries. These ones were spiked with two multi-residue solutions containing 240 active substances in methanol and 155 active substances in acetone. Solutions were prepared in an accredited ISO17025 laboratory (ISP chemical residues and contaminants) according to an accredited internal procedure. Stability and variability tests were passed according to the quality criteria of the SANTE/11945/2015 document [32]. All the solutions (individual, intermediate mix, and spiking) were stocked and aliquoted at −20 °C. The spiking was done by spraying small droplets of 250 µL and 100 µL of the two solutions (concentration 2 µg/mL and 5 µg/mL respectively) using a 250 µL and 100 µL calibrated syringes, respectively. No pesticides were detected in the three blanco samples, proving an absence of pesticides in the gloves themselves. The spiking was calculated to reach an average concentration of about 0.1 mg/kg gloves for each active substance. Twenty-four hours after deposit, the samples were cut into small pieces and stored in freezing bags at −18 °C until analysis. The recoveries were calculated using statistical analysis software developed by Verplaetse in 1998, modified by Van Loco in 2003 [33]. The preliminary study allowed a percentage of recovery for 395 substances to be determined, but the recoveries of nine active substances (azadirachtin, captan, cyflumetofen, etoxazole, fluoxastrobin, pymetrozine, spinetoram, tetrahydrophtalimide, and thiophanate methyl) that were not available in the spiking solutions could not been determined.

### 2.4. Florists Exposure Assessment Calculation

Classically, exposure is described as the amount of an agent that contacts the outer boundary of the body. However, this definition of exposure is limited because the real interest in risk assessment is the amount of an agent that breeches the outer boundary of the body (dose) and is capable of being distributed to one or more organs to exert a toxic effect (target dose) [34]. For dermal exposure to occur, an individual must have contact with the chemical in a given medium. The amount of exposure will depend on the concentration of the chemical contacting a given area of skin—the dermal loading or skin adherence, the ability of the chemical to penetrate and pass through intact skin—the dermal dose, and the duration and frequency of contact in terms of the intervals of contact and the number of intervals per day, weeks, months, or even a lifetime [34].

The exposure of workers can be estimated for activities that involve contact with treated crops or products. The main route of exposure for florists who handle daily cut flowers and ornamentals is skin contact and subsequent dermal absorption.

The potential dermal exposure (PDE) values were estimated as the amount of pesticide residues with low adhesion that were transferred from flowers to gloves. For each active substance, PDE was calculated as follows:

PDE (in mg a.s./kg bw per day) = ((C (mg/kg) × GW (kg)) × T (h)) × 3)/bw (kg)

where C is the concentration of active substance in the sub-sample (5 g), GW is the average weight of the cotton gloves samples (57 g + 0.17 g), T is the task duration during the trial (2 h), and bw is the body weight (60 kg).

A total task duration value of 6 h/day was used to assess the dermal exposure of florists. A recent survey in Belgium [4] showed that 60% of the florists worked between 6 and 7 h/day. The time spent preparing bouquets and handling flowers vary greatly over the year, but is always quite high, varying on average from 2 to 6 h/day for 80% of the florists in the low season and for 40% of the florists in the high season. This handling time could be in excess of 6 h for 8% of the florists in the low season, but during the high season or special occasions, an intense working period, 60% spent more than 6 h/day on this work. Only 12% of the florists worked less than 2 h/day in the low season. A default body weight (bw) value of 60 kg is used in line with the recent EFSA Guidance Document to cover a range of professionally exposed adults [15].

The PDE values were then converted into systemic doses using an appropriate dermal absorption percentage of 75% (default value) [35]. To obtain the actual dermal exposure (ADE), the potential dermal exposure (PDE) values in absence of protection can be reduced by 90%, the penetration factor being equal to 10% when workwear and gloves are worn [15].

The risk characterisation is obtained as the ratio of the exposure level to the reference value of each active substance, the AOEL (Acceptable Operator Exposure Level; in mg a.s./kg·bw per day), which should not be exceeded to avoid any detrimental effect on florists’ health. Several prediction levels of the PDE were considered, including the mean, 75th percentile, 90th percentile, and the maximum (in mg/kg bw per day) to assess the risk for florists. Therefore, the systemic exposure (SE) values (mean, 75th percentile, 90th percentile, and maximum) were expressed as percentage of the AOEL. It has been assumed that the most appropriate level to cover and assess the risk is the maximum value of the SE (SEmax or worst case).

## 3. Results

### 3.1. Pesticide Residues Identified on Glove Samples

All glove samples appeared to be contaminated by high levels of pesticide residues for most active substances. A total of 111 a.s. were identified, with an average of about 37 a.s./sample and an average total pesticide residue concentrations per glove sample of 22.22 mg/kg (Table 1). Fourteen active substances (azoxystrobine (80%), benomyl (95%), boscalid (90%), clofentezine (90%), fenhexamid (85%), flonicamid (90%), fludioxonil (85%), fluopyram (80%), imidacloprid (75%), iprodione (95%), lufenuron (90%), methiocarb (75%), procymidone (85%), and spiroxamine (80%)) are the most frequently detected. They are present on more than 15 of the 20 samples (75%).

### 3.2. Pesticide Residues Hazard Characterisation

The intrinsic toxicological properties (acute and chronic toxicity including mutagenic, carcinogenic and reproductive hazards) of each substance identified on the gloves were collected in pesticide databases (European Union Pesticides Database, Directorate-General for Health and Food Safety) and JMPR (Joint Meeting on Pesticide Residues)reports (FAO (Food and Agriculture Organization) Joint Meeting on Pesticide Residues) (Table 2) [28,36,37,38,39].

### 3.3. Florists Exposure Assessment

Table 3 presents the number of detection (N), the Potential Dermal Exposure (mean, 75th percentile, 90th percentile, and maximum values) in mg/kg bw per day, and the systemic exposure as a percentage of the AOEL calculated for SE (mean, 75th percentile, 90th percentile, and maximum values), for all active substances detected on the gloves of florists. All values exceeding 100% of the AOEL indicate a potential risk situation.

The actual dermal exposure (ADE) values have been calculated for the same prediction levels of risk. None of these values exceed the AOEL. Therefore the detailed results were not reported here.

### 3.4. Most Frequently Detected Active Substances and AOEL Exceedance

**Figure 1:**
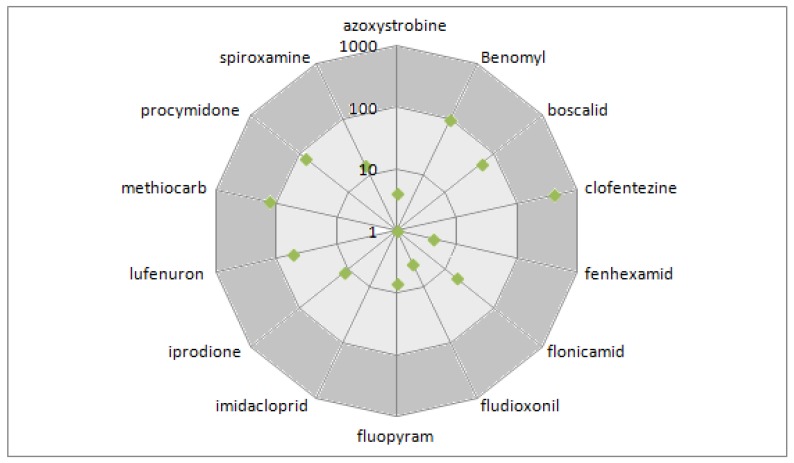
The maximum systemic exposure (SE_MAX_) of the fourteen most frequently detected active substances on gloves worn by florists as a percentage of the Acceptable Operator Exposure Level (AOEL), Green Symbol: SE_MAX_ as a percentage of the AOEL.

Fourteen active substances were the most frequently detected in the glove samples (frequency > 75%). Therefore, it was considered that a great part of the risk could be related to the repeated exposure of the florists to these specific 14 substances. Figure 1 presents the percentage of their respective AOEL (from 0 to more than 100% AOEL) for their SE_MAX_ values.

## 4. Discussion

A linear relationship exists between the levels of dislodgeable residue and the dermal exposure [40,41,42]. Contact with contaminated flowers resulted in the transfer of pesticide residues to gloves worn by florists. All glove samples appeared to be highly contaminated by many different pesticide residues (111 active substances detected with an average of about 37 active substances per sample and a total concentration per glove sample of 22.22 mg/kg). These concentrations are 1000 times higher than the concentrations which are usually detected on foodstuffs. Half of the detected active substances are insecticides and the other half are fungicides. Only one substance is a growth regulator (paclobutrazol) and another one is a herbicide (simazine). Three fungicides (benomyl and its metabolite carbendazim, boscalid, and iprodione) and three insecticides (clofentezine, lufenuron, and flonicamid) are the most frequently detected active substances (90% of the samples). Twenty eight active substances (25%) are detected only once.

The maximum residue concentrations are measured for boscalid, clofentezine, iprodione, and mandipropamid (26.21, 18.37, 16.93, and 16.50 mg/kg, respectively). Boscalid, novaluron, clofentezine, iprodione, and spirodiclofen present the highest average concentrations, with 3.47 and 3.38, 2.88, 2.42, and 2 mg/kg, respectively. Boscalid is the active substance that has both the highest average and maximum concentrations out of all the active substances analysed.

Of the 111 detected active substances, most of the pesticides belong to the following chemical groups: triazoles (13 a.s.); pyrethroids (8 a.s.); organophosphates (7 a.s.); carbamates and strobilurins (6 a.s.); and benzoylurea, keto-enol, and neonicotinoids (4 a.s.). Pesticides from these families are known for their toxicological properties (acute toxicity, with an action on the nervous system). Many active substances detected in the glove samples may affect the skin of the florists after exposure by contact (allergic reaction: 23; skin irritation: 5; harmful in contact with skin: 5; severe skin burns and eye damage: 1; toxic in contact with skin: 1). However, there are no active substances that could be fatal in contact with skin. According to the CLP (classification, labelling, and packaging) classification (Table 2), some of these active substances have potential hazardous chronic effects. Seven active substances are suspected of damaging fertility or the unborn child, two may damage fertility or the unborn child, one may cause genetic defects, one is suspected of causing genetic defects, and another one may cause harm to breast-fed children. Moreover, ten active substances are also suspected of causing cancer after prolonged or repeated exposure. The potential health effects of these hazards are in accordance with symptoms recorded in various publications. In the survey of Morse et al. [3] the symptoms most frequently reported by the florists after exposure are headaches (20%), skin irritation (20%), and watery eyes (20%). In the survey of Restrepo et al. [13], a moderate increase in the prevalence of abortion, prematurity, and congenital malformations for pregnant female workers in floriculture was noted. During the interviews conducted by Toumi et al. [4], only one florist mentioned frequent headaches and recurrent tiredness, but many of them declared suffering from various symptoms like skin allergy, eye irritation, itching of their skin, respiratory problems, thyroid problems and even, for two out of 25, cancer. These testimonies should of course be considered with caution when no diagnosis can support such declarations. Based on repeated observations of workers who are regularly exposed to pesticides at concentrations above their AOEL, such exposures can result in adverse health effects.

The potential dermal exposures of florists were estimated for the average, for different percentiles, and for the maximum concentration of residues in samples (Table 3). The results from the different percentiles used to estimate PDE vary by orders of magnitude. As was shown in Table 3, no active substance exceeds the AOEL for PDEmean and PDE P75 values. However, at the P90 and at the maximum (or worst case) values of PDE, three and five active substances respectively exceed the AOEL indicating risk situations. The potential dermal exposure values are in accordance with Thongsinthusak et al. [43] and Brouwer et al. [29]. They have respectively reported contamination levels of 0.0005 mg/kg bw per day after handling chrysanthemums and roses and 0.1714 mg/kg bw per day during cutting, sorting, and bundling of roses. For a vast majority of active substances, PDE values obtained in our trial have the same order of magnitude. Even for the worst cases of exposure (e.g., clofentezine or methiocarb), the values are rather similar to previously reported data. Only results of dermal exposure reported by Brouwer et al. [17] for workers in contact with flowers at the field were significantly higher. Average exposures of 0.8571 mg/kg bw per day during cutting and 0.6000 mg/kg bw per day during sorting and bundling of carnations were observed. The short elapsed time between application of pesticides and the re-entry of workers as well as the application rates and the nature of their activities could explain a higher transfer of residues after contact. Nevertheless, the comparison between those previous studies and our results should be considered with caution as local situations are different and practises have evolved with time.

For the systemic exposure, one active substance (clofentezine) exceeds the AOEL at the P90 predictive level. In the worst case, SE_MAX_ (at the maximum concentrations), four active substances (clofentezine, famoxadone, methiocarb, and pyridaben) exceed their respective AOEL values. Among the 14 most frequently detected active substances, two have SE_MAX_ values exceeding the AOEL. Exposure could be particularly critical for clofentezine with SE_MAX_ values that are four times higher the AOEL (393%).

For the actual dermal exposure (ADE), whatever the PDE values considered, it is interesting to confirm that no active substances exceed the AOEL when the florists are wearing PPE. A few studies on workers have confirmed that protective clothing [44,45] and gloves [46] can reduce the amount of pesticides reaching the skin. It is assumed today that their potential dermal exposure can be reduced by 90% when workers protect themselves with appropriate PPE [15]. Nevertheless, the survey conducted in Belgium [4] showed that this scenario is not representative of their habits; the majority of florists do not wear gloves, or any other PPE, even if they spend 2 to 6 h per day handling cut flowers and preparing bouquets.

## 5. Conclusions

In conclusion, the exposure of florists is an example of a unique situation in which a professional is exposed regularly to both a very high number of toxic chemicals and rather high concentration levels. According to the results of the risk assessment, Belgian florists who handle a large number of flowers are at risk of exposure to pesticides residues with potential effects on their health. To better assess the risk, bio-monitoring of the florists with analysis of their blood, urines, and hairs is still to be investigated.

Meanwhile, to reduce their exposure, solutions could be recommended. The priority should be to raise the level of awareness among the florists who can change their habits and practices if they want to minimize their exposure. Wearing gloves, washing their hands and their arms, and respecting hygiene rules could be effective. In the near future, it is necessary to promote a better pesticide management at the field level (integrated pest management, certification schemes, and labels) or even organic flower production if clients are ready to pay. Moreover, extending the European regulation on maximum residue limits (Regulation (EC) N°396/2005) for pesticide residues on flowers or controlling the residue levels on cut flowers could also be discussed.

## Figures and Tables

**Table 1:** Total number of active substances (a.s.) detected and total pesticide residue concentrations (mg/kg) in 20 samples of gloves.

Samples from Florists (2 Pairs of Gloves/Sample)	Total Number of Active Substances Detected (LOQ < 0.01 mg/kg)	Total Pesticide Residues Concentrations (mg/kg) *
Sample N°1	47	113.44
Sample N°2	48	24.75
Sample N°3	43	5.69
Sample N°4	54	31.23
Sample N°5	68	70.41
Sample N°6	40	8.88
Sample N°7	19	5.08
Sample N°8	59	41.54
Sample N°9	24	13.92
Sample N°10	12	1.43
Sample N°11	40	24.07
Sample N°12	31	7.35
Sample N°13	51	28.83
Sample N°14	23	7.88
Sample N°15	52	36.18
Sample N°16	41	9.38
Sample N°17	21	1.36
Sample N°18	35	6.85
Sample N°19	20	4.50
Sample N°20	20	1.62
Mean	37.40	22.22
Median	40.00	8.11

* All the active substances below the limit of quantification (LOQ) were not taken into account in the sum.

**Table 2:** Alphabetic classification of all a.s. detected in the 20 samples of gloves with their chemical family, biological activity, detection frequency (% samples where the a.s. is detected), average (±SD), and range of concentrations (mg/kg gloves, LOQ < 0.01 mg/kg) in the samples and their toxicological properties (Dermal LD50, AOEL values, and CLP (Classification, Labelling and Packaging) Classification according the EU Pesticides database).

Active Substance	Chemical Family	Biological Activity	Detection Frequency (%)	Average Concentration (mg/kg) ± SD	Range (mg/kg)	Dermal LD_50_ (mg/kg bw)	AOEL Values (mg/kg bw/day)	CLP Classification
Acephate *	Organophosphates	I	10%	0.055 ± 0.055	<LOQ-0.094	>10,000	0.0300	H302
Acetamiprid	Neonicotinoids	I	30%	0.219 ± 0.263	<LOQ-0.602	>2000	0.0700	H302
Acrinathrin	Pyrethroids	I	10%	0.285 ± 0.372	<LOQ-0.547	>2000	0.0070	-
Ametoctradin	Triazolopyrimidine	F	55%	0.859 ± 1.921	<LOQ-6.477	>2000	2.0000	-
Azadirachtin *	limonoid	I	20%	0.169 ± 0.137	<LOQ-0.350	>2000	0.1000	-
Azoxystrobine	Strobilurins	F	80%	0.617 ± 0.917	<LOQ-3.287	>2000	0.2000	H331
Benomyl (carbendazim) *	Benzimidazole	F	95%	0.739 ± 2.040	<LOQ-9.000	>10,000	0.0200	H315, H317, H335, H340, H360
Bifenazate *	Carbazates	I	15%	0.132 ± 0.164	<LOQ-0.320	>5000	0.0028	H317, H373
Bifenthrin	Pyrethroids	I	15%	0.059 ± 0.048	<LOQ-0.108	>2000	0.0075	H300, H317, H331 H351, H372
Bitertanol	Triazoles	F	20%	0.043 ± 0.038	<LOQ-0.097	>5000	0.0100	-
Boscalid	Carboxamides	F	90%	3.471 ± 6.810	<LOQ-26.213	>2000	0.1000	-
Bupirimate	Pyrimidine	F	35%	0.170 ± 0.184	<LOQ-0.565	>2000	0.0500	-
Buprofezin	Thiadiazine	I	35%	0.488 ± 1.093	<LOQ-2.963	1635–3847	0.0400	-
Captan *	Phthalimides	F	5%	0.510 ± 0.240	<LOQ-0.680	>2000	0.1000	H317, H318, H331 H351
Carbofuran	Carbamates	I	5%	0.012 ± 0.000	<LOQ-0.012	>500	0.0003	H300, H330
Chlorantraniliprole	Diamides	I	15%	0.215 ± 0.878	<LOQ-0.373	>5000	0.3600	H301
Chlorothalonil *	Organochlorine	F	25%	0.197 ± 0.190	<LOQ-0.420	>5000	0.3600	H317, H318, H330 H335, H351
Chlorpyrifos (-ethyl)	Organophosphates	I	10%	0.041 ± 0.012	<LOQ-0.049	>2000	0.0010	H301
Clofentezine	Quinoxalines	I	90%	2.881 ± 5.604	<LOQ-18.373	>2100	0.0100	-
Cyflumetofen *	Benzoylacetonitriles	I	60%	0.116 ± 0.204	<LOQ-0.750	>5000	0.1100	-
Cyhalothrin	Pyrethroids	I	35%	0.107 ± 0.158	<LOQ-0.452	632	0.0025	-
Cypermethrin	Pyrethroids	I	50%	0.135 ± 0.161	<LOQ-0.455	>1600	0.0600	H302, H332, H335
Cyproconazole	Triazoles	F	5%	0.043 ± 0.000	<LOQ-0.043	>2000	0.0200	H302, H361
Cyprodinil	Anilinopyrimidines	F	70%	0.132 ± 0.219	<LOQ-0.745	>2000	0.0300	H317
Deet		I	85%	0.146 ± 0.078	<LOQ-0.299	-	-	-
Deltamethrin	Pyrethroids	I	40%	0.074 ± 0.066	<LOQ-0.221	>2000	0.0075	H301, H331
Dicofol	Organochlorine	I	5%	0.035 ± 0.000	<LOQ-0.035	>5000	-	H302, H312, H315, H317
Difenoconazole	Triazoles	F	50%	0.120 ± 0.175	<LOQ-0.552	2010	0.1600	-
Diflubenzuron	Benzoylureas	I	10%	0.036 ± 0.021	<LOQ-0.051	>10,000	0.0330	-
Dimethoate	Organophosphates	I	5%	0.016 ± 0.000	<LOQ-0.016	>7000	0.0010	H302, H312
Dimethomorph	Cinnamic acid	F	70%	0.476 ± 0.878	<LOQ-3.485	>2000	0.1500	-
Diphenylamine	Amides	F	25%	0.144 ± 0.139	<LOQ-0.392	1700	0.1000	H315, H317
Dodemorph *	Morpholine	F	65%	0.503 ± 1.017	<LOQ-3.700	>2000	0.0330	H314, H317, H361, H373
Endosulfan	Organochlorine	I	25%	0.092 ± 0.071	<LOQ-0.183	500	-	H300, H312, H330
Etoxazole *	Oxazolines	I	45%	0.301 ± 0.552	<LOQ-1.700	>2000	0.0300	-
Famoxadone	Oxazolidinediones	F	60%	0.563 ± 0.779	<LOQ-2.627	>2000	0.0048	H373
Fenamidone	Imidazolinones	F	40%	0.215 ± 0.366	<LOQ-1.056	>2000	0.3000	-
Fenazaquin	Quinazolines	I	10%	0.689 ± 1.039	<LOQ-1.364	>2000	0.0100	H301, H332
Fenhexamid	Phenylpyrroles	F	85%	1.052 ± 1.713	<LOQ-5.195	>5000	0.3000	-
Fenoxycarb	Carbamates	I	5%	0.047 ± 0.000	<LOQ-0.047	>2000	0.1000	H351
Fenpyroximate	Pyridazinones	I	5%	1.268 ± 0.000	<LOQ-1.268	>2000	0.0050	H301, H317, H330
Fenvalerate	Pyrethroids	I	10%	0.198 ± 0.201	<LOQ-0.339	5000	-	-
Fipronil	Phenylpyrazoles	I	40%	0.281 ± 0.407	<LOQ-1.199	>2000	0.0035	H301, H311, H331, H372
Flonicamid	Pyridinecarboxamides	I	90%	0.379 ± 0.563	<LOQ-1.964	>5000	0.0250	H302
Fluazinam	Phenylpyridylamines	F	20%	0.098 ± 0.069	<LOQ-0.197	5500	0.0040	H317, H318, H332, H361
Flubendiamide	Keto-Enol	I	15%	0.128 ± 0.164	<LOQ-0.317	>2000	0.0060	-
Fludioxonil	Phenylpyrroles	F	85%	1.665 ± 3.125	<LOQ-12.278	>2000	0.5900	-
Flufenoxuron	Benzoyl urea	I	15%	0.317 ± 0.385	<LOQ-0.762	>2000	0.0100	H362
Fluopicolide	Acylpicolides	F	30%	0.257 ± 0.430	<LOQ-1.100	>5000	0.0500	-
Fluopyram	Pyridines	F	80%	0.360 ± 0.428	<LOQ-1.624	>2000	0.0500	-
Fluoxastrobin *	Strobilurins	F	5%	0.031 ± 0.000	<LOQ-0.031	>2000	0.0300	-
Flusilazole	Triazoles	F	5%	0.631 ± 0.000	<LOQ-0.631	>2000	0.0050	H302, H351, H360
Flutolanil	Phenylamides	F	5%	0.207 ± 0.000	<LOQ-0.207	>5000	0.5600	-
Flutriafol	Triazoles	F	20%	0.107 ± 0.173	<LOQ-0.365	>2000	0.0500	-
Fluxapyroxad	Pyrazole carboxamides	F	15%	0.147 ± 0.119	<LOQ-0.242	>2000	0.0400	-
Hexythiazox	Carboxamides	I	55%	0.344 ± 0.840	<LOQ-2.854	>5000	0.0090	-
Imidacloprid	Neonicotinoids	I	75%	0.072 ± 0.046	<LOQ-0.203	>5000	0.0800	H302
Indoxacarb	Oxadiazines	I	40%	0.078 ± 0.104	<LOQ-0.322	>5000	0.0040	H301, H317, H332, H372
Iprodione	Dicarboximides	F	95%	2.422 ± 3.969	<LOQ-16.931	>2000	0.3000	H351
Iprovalicarb	Carbamates	F	40%	0.236 ± 0.367	<LOQ-1.085	>5000	0.0150	-
Kresoxim-methyl	Strobilurins	F	50%	0.589 ± 1.097	<LOQ-3.471	>2000	0.9000	H351
Lufenuron	Benzoylureas	I	90%	0.289 ± 0.557	<LOQ-2.462	>2000	0.0100	H317
Malathion	Organophosphates	I	5%	0.018 ± 0.000	<LOQ-0.018	>2000	-	H302, H317
Mandipropamid	Mandelic acid	F	60%	2.272 ± 6.804	<LOQ-23.836	>5000	0.1700	-
Mepanipyrim	Anilinopyrimidines	F	20%	0.217 ± 0.234	<LOQ-0.470	>2000	0.0700	H351
Metalaxyl (metalaxyl-M)	Acylamines	F	15%	0.348 ± 0.457	<LOQ-0.875	>3100	0.0800	H302, H317, H302, H318
Methiocarb	Carbamates	I	75%	1.209 ± 2.387	<LOQ-7.664	>5000	0.0130	H301
Methoxyfenozide	Diacylhydrazines	F	70%	0.164 ± 0.180	<LOQ-0.676	>5000	0.1000	-
Metrafenone	Benzophenones	F	15%	0.086 ± 0.064	<LOQ-0.139	>5000	0.4300	-
Myclobutanil	Triazoles	F	20%	0.068 ± 0.030	<LOQ-0.097	>5000	0.0300	H302, H319, H361
Nitrothal-isopropyl	-	F	5%	0.087 ± 0.000	<LOQ-0.087	>5000	-	-
Novaluron	Benzoylureas	I	5%	3.382 ± 0.000	<LOQ-3.382	>2000	-	-
Oxycarboxin	Anilides	F	5%	0.017 ± 0.000	<LOQ-0.017	16,000	-	H302
Paclobutrazol	Triazoles	R	15%	0.291 ± 0.451	<LOQ-0.811	>1000	0.1000	-
Penconazole	Triazoles	F	5%	0.131 ± 0.000	<LOQ-0.131	>3000	0.0300	H302, H361
Permethrin	Pyrethroids	I	5%	0.037 ± 0.000	<LOQ-0.037	>2000	-	H302, H332, H335
Picoxystrobin	Strobilurins	F	25%	0.223 ± 0.451	<LOQ-1.030	>2000	0.0430	-
Piperonyl-butoxyde	-	I	45%	0.065 ± 0.111	<LOQ-0.351	>2000	-	-
Pirimicarb	Carbamates	I	15%	0.023 ± 0.012	<LOQ-0.039	>2000	0.0350	H301
Pirimiphos-methyl	Organophosphates	I	5%	0.024 ± 0.000	<LOQ-0.024	>2000	0.0200	H302
Prochloraz	Imidazoles	F	65%	0.476 ± 0.837	<LOQ-3.049	>2000	0.0200	H302
Procymidone	Dicarboximides	F	85%	0.729 ± 1.352	<LOQ-4.207	>2500	0.0120	-
Profenofos	Organophosphates	I	5%	0.013 ± 0.000	<LOQ-0.013	>2000	-	H302, H312, H332
Propamocarb *	Carbamates	F	65%	0.770 ± 1.462	<LOQ-5.100	>2000	0.2900	-
Propiconazole	Triazoles	F	20%	0.032 ± 0.022	<LOQ-0.064	>4000	0.1000	H302, H317
Pymetrozine *	Pyridine-azométhrine	I	45%	0.072 ± 0.084	<LOQ-0.280	>2000	0.0300	H351
Pyraclostrobin	Strobilurins	F	50%	0.589 ± 0.845	<LOQ-2.222	>2000	0.0150	H315, H331
Pyridaben	Pyridazinones	I	30%	0.654 ± 1.103	<LOQ-2.804	>2000	0.0050	H301, H331
Pyridalyl	Dihalopropenes	I	65%	0.175 ± 0.177	<LOQ-0.625	>5000	0.0200	-
Pyrimethanil	Anilinopyrimidines	F	25%	0.094 ± 0.111	<LOQ-0.283	>5000	0.1200	-
Pyriproxyfen	Pyridines	I	5%	0.015 ± 0.000	<LOQ-0.015	>2000	0.0400	-
Simazine	Triazines	H	5%	0.021 ± 0.000	<LOQ-0.021	-	-	H351
Spinetoram *	Spinosyns	I	25%	0.018 ± 0.005	<LOQ-0.024	>5000	0.0065	-
Spinosad *	Spinosyns	I	35%	0.149 ± 0.306	<LOQ-0.840	>5000	0.0120	-
Spirodiclofen *	Keto-Enol	I	5%	2.000 ± 0.000	<LOQ-2.000	>2000	0.0090	-
Spiromesifen	Keto-Enol	I	5%	0.025 ± 0.000	<LOQ-0.025	>2000	0.0150	-
Spirotetramat *	Keto-Enol	I	30%	0.072 ± 0.047	<LOQ-0.140	>2000	0.0500	H317, H319, H335, H361
Spiroxamine	Spirocétalamines	F	80%	0.363 ± 0.323	<LOQ-1.031	1068	0.0150	H302, H312, H315, H317, H332
Tebuconazole	Triazoles	F	40%	0.323 ± 0.511	<LOQ-1.494	>2000	0.0300	H302, H361
Tebufenozide	Diacylhydrazines	I	5%	0.155 ± 0.000	<LOQ-0.155	>5000	0.0080	-
Tebufenpyrad	Pyrazoles	I	10%	0.161 ± 0.197	<LOQ-0.300	>2000	0.0100	H301, H317, H332, H373
Tetraconazole	Triazoles	F	5%	0.037 ± 0.000	<LOQ-0.037	-	0.0300	H302, H332
Tetramethrine *	Pyrethroids	I	5%	0.020 ± 0.000	<LOQ-0.020	-	-	-
Thiabendazole *	Benzimidazoles	F	10%	0.042 ± 0.020	<LOQ-0.056	>5000	0.1000	-
Thiacloprid	Neonicotinoids	I	45%	0.347 ± 0.596	<LOQ-1.777	>2000	0.0200	-
Thiametoxam	Neonicotinoids	I	55%	0.294 ± 0.360	<LOQ-1.031	>2000	0.0800	H302
Thiophanate methyl *	Benzimidazoles	F	35%	0.087 ± 0.083	<LOQ-0.230	>10,000	0.0800	H317, H332, H341
Tolclofos-methyl	Organophosphates	F	20%	0.035 ± 0.017	<LOQ-0.057	>5000	0.2000	H317
Triadimefon (triadimenol)	Triazoles	F	5%	0.053 ± 0.000	<LOQ-0.053	>5000	0.0500	H302, H317
Trifloxystrobin	Strobilurins	F	50%	0.161 ± 0.169	<LOQ-0.529	>2000	0.0600	H317
Triflumizole	Triazoles	F	5%	0.027 ± 0.000	<LOQ-0.027	>5000	0.0500	-

H300: Fatal if swallowed; H301: Toxic if swallowed; H302: Harmful if swallowed;H311: Toxic in contact with skin; H312: Harmful in contact with skin; H314: Causes severe skin burns and eye damage; H315: Causes skin irritation; H317: May cause an allergic skin reaction; H318: Causes serious eye damage; H319: Causes serious eye irritation; H330: Fatal if inhaled; H331: Toxic if inhaled; H332: Harmful if inhaled; H335: May cause respiratory irritation; H340: May cause genetic defects; H341: Suspected of causing genetic defects; H351: Suspected of causing cancer; H360: May damage fertility or the unborn child; H361: Suspected of damaging fertility or the unborn child; H362: May cause harm to breast-fed children; H372: Causes damage to organs through prolonged or repeated exposure; H373: May cause damage to organs through prolonged or repeated exposure; F: Fungicide; H: Herbicide; I: Insecticide; R: Growth regulator; *: active substances with non-corrected recovery due to recovery below or above 50–130%.

**Table 3:** All a.s. present in the 20 samples of gloves and the corresponding calculation: number of detection (N), potential dermal exposure (mean, 75th percentile, 90th percentile, and maximum values) in mg/kg bw per day and the systemic exposure as a percentage of the AOEL calculated for SE (mean, 75th percentile, 90th percentile, and maximum values) for all active substances detected on the gloves of florists (*: Value exceeds the AOEL).

Active Substance	N	PDE (Mean) (mg/kg bw per day)	SE (Mean) in % of AOEL	PDE (75th P) (mg/kg bw per day)	SE (75th P) in % of AOEL	PDE (90th P) (mg/kg bw per day)	SE (90th P) in % of AOEL	PDE (Maximum) (mg/kg bw per day)	SE (Maximum) in % of AOEL
Acephate	2	0.00016	0%	0.00021	1%	0.00025	1%	0.00027	1%
Acetamiprid	6	0.00062	1%	0.00115	1%	0.00158	2%	0.00172	2%
Acrinathrin	2	0.00081	9%	0.00118	13%	0.00141	15%	0.00156	17%
Ametoctradin	11	0.00245	0%	0.00181	0%	0.00389	0%	0.01846	0%
Azadirachtin	4	0.00048	0%	0.00068	1%	0.00087	1%	0.00100	1%
Azoxystrobine	16	0.00176	1%	0.00260	1%	0.00466	2%	0.00937	4%
Benomyl (carbendazim)	19	0.00210	8%	0.00168	6%	0.00342	13%	0.02565 *	96%
Bifenazate	3	0.00038	10%	0.00053	14%	0.00076	20%	0.00091	24%
Bifenthrin	3	0.00017	2%	0.00023	2%	0.00028	3%	0.00031	3%
Bitertanol	4	0.00012	1%	0.00016	1%	0.00023	2%	0.00028	2%
Boscalid	18	0.00989	7%	0.00943	7%	0.02767	21%	0.07471	56%
Bupirimate	7	0.00048	1%	0.00049	1%	0.00096	1%	0.00161	2%
Buprofezin	7	0.00139	3%	0.00040	1%	0.00370	7%	0.00845	16%
Captan	1	0.00145	1%	0.00170	1%	0.00184	1%	0.00194	1%
Carbofuran	1	0.00003	8%	0.00003	8%	0.00003	8%	0.00003	8%
Chlorantraniliprole	3	0.00061	0%	0.00087	0%	0.00099	0%	0.00106	0%
Chlorothalonil	5	0.00056	0%	0.00105	0%	0.00114	0%	0.00120	0%
Chlorpyrifos (-ethyl)	2	0.00012	9%	0.00013	10%	0.00014	11%	0.00014	11%
Clofentezine	18	0.00821	62%	0.00623	47%	0.02862 *	215%	0.05236 *	393%
Cyflumetofen	12	0.00033	0%	0.00029	0%	0.00042	0%	0.00214	1%
Cyhalothrin	7	0.00030	9%	0.00029	9%	0.00074	22%	0.00129	39%
Cypermethrin	10	0.00039	0%	0.00074	1%	0.00093	1%	0.00130	2%
Cyproconazole	1	0.00012	0%	0.00012	0%	0.00012	0%	0.00012	0%
Cyprodinil	14	0.00038	1%	0.00033	1%	0.00112	3%	0.00212	5%
Deet	17	0.00042	-	0.00054	-	0.00068	-	0.00085	-
Deltamethrin	8	0.00021	2%	0.00023	2%	0.00040	4%	0.00063	6%
Dicofol	1	0.00010	-	0.00010	-	0.00010	-	0.00010	-
Difenoconazole	10	0.00034	0%	0.00041	0%	0.00085	0%	0.00157	1%
Diflubenzuron	2	0.00010	0%	0.00012	0%	0.00014	0%	0.00014	0%
Dimethoate	1	0.00005	4%	0.00005	4%	0.00005	4%	0.00005	4%
Dimethomorph	14	0.00136	1%	0.00090	0%	0.00158	1%	0.00993	5%
Diphenylamine	5	0.00041	0%	0.00026	0%	0.00078	1%	0.00112	1%
Dodemorph	13	0.00143	3%	0.00086	2%	0.00295	7%	0.01055	24%
Endosulfan	5	0.00026	-	0.00043	-	0.00049	-	0.00052	-
Etoxazole	9	0.00086	2%	0.00108	3%	0.00202	5%	0.00485	12%
Famoxadone	12	0.00160	25%	0.00167	26%	0.00412	64%	0.00749 *	117%
Fenamidone	8	0.00061	0%	0.00044	0%	0.00175	0%	0.00301	1%
Fenazaquin	2	0.00214	16%	0.00319	24%	0.00381	29%	0.00423	32%
Fenhexamid	17	0.00300	1%	0.00261	1%	0.01058	3%	0.01483	4%
Fenoxycarb	1	0.00014	0%	0.00014	0%	0.00014	0%	0.00014	0%
Fenpyroximate	1	0.00361	54%	0.00361	54%	0.00361	54%	0.00361	54%
Fenvalerate	2	0.00056	-	0.00077	-	0.00089	-	0.00097	-
Fipronil	8	0.00080	17%	0.00082	18%	0.00203	44%	0.00342	73%
Flonicamid	18	0.00108	3%	0.00140	4%	0.00358	11%	0.00560	17%
Fluazinam	4	0.00028	5%	0.00032	6%	0.00046	9%	0.00056	11%
Flubendiamide	3	0.00036	5%	0.00051	6%	0.00075	9%	0.00090	11%
Fludioxonil	17	0.00474	1%	0.00541	1%	0.01162	1%	0.03499	4%
Flufenoxuron	3	0.00090	7%	0.00124	9%	0.00180	14%	0.00217	16%
Fluopicolide	6	0.00073	1%	0.00073	1%	0.00204	3%	0.00314	5%
Fluopyram	16	0.00103	2%	0.00126	2%	0.00228	3%	0.00463	7%
Fluoxastrobin	1	0.00009	0%	0.00009	0%	0.00009	0%	0.00009	0%
Flusilazole	1	0.00180	27%	0.00180	27%	0.00180	27%	0.00180	27%
Flutolanil	1	0.00059	0%	0.00059	0%	0.00059	0%	0.00059	0%
Flutriafol	4	0.00030	0%	0.00031	0%	0.00075	1%	0.00104	2%
Fluxapyroxad	3	0.00042	1%	0.00061	1%	0.00066	1%	0.00069	1%
Hexythiazox	11	0.00098	8%	0.00055	5%	0.00103	9%	0.00813	68%
Imidacloprid	15	0.00021	0%	0.00025	0%	0.00031	0%	0.00058	1%
Indoxacarb	8	0.00022	4%	0.00018	3%	0.00050	9%	0.00092	17%
Iprodione	19	0.00690	2%	0.00550	1%	0.01569	4%	0.04825	12%
Iprovalicarb	8	0.00067	3%	0.00080	4%	0.00169	8%	0.00309	15%
Kresoxim-methyl	10	0.00168	0%	0.00135	0%	0.00449	0%	0.00989	1%
Lufenuron	18	0.00082	6%	0.00064	5%	0.00125	9%	0.00702	53%
Malathion	1	0.00005	-	0.00005	-	0.00005	-	0.00005	-
Mandipropamid	12	0.00647	3%	0.00127	1%	0.00395	2%	0.06793	30%
Mepanipyrim	4	0.00062	1%	0.00111	1%	0.00125	1%	0.00134	1%
Metalaxyl (metalaxyl-M)	3	0.00099	1%	0.00140	1%	0.00205	2%	0.00249	2%
Methiocarb	15	0.00345	20%	0.00176	10%	0.01306 *	75%	0.02184 *	126%
Methoxyfenozide	14	0.00047	0%	0.00063	0%	0.00088	1%	0.00193	1%
Metrafenone	3	0.00025	0%	0.00035	0%	0.00038	0%	0.00040	0%
Myclobutanil	4	0.00019	0%	0.00025	1%	0.00027	1%	0.00028	1%
Nitrothal-isopropyl	1	0.00025	-	0.00025	-	0.00025	-	0.00025	-
Novaluron	1	0.00964	-	0.00964	-	0.00964	-	0.00964	-
Oxycarboxin	1	0.00005	-	0.00005	-	0.00005	-	0.00005	-
Paclobutrazol	3	0.00083	1%	0.00120	1%	0.00187	1%	0.00231	2%
Penconazole	1	0.00037	1%	0.00037	1%	0.00037	1%	0.00037	1%
Permethrin	1	0.00011	-	0.00011	-	0.00011	-	0.00011	-
Picoxystrobin	5	0.00063	1%	0.00009	0%	0.00180	3%	0.00294	5%
Piperonyl-butoxyde	9	0.00019	-	0.00016	-	0.00042	-	0.00100	-
Pirimicarb	3	0.00008	0%	0.00010	0%	0.00010	0%	0.00011	0%
Pirimiphos-methyl	1	0.00007	0%	0.00007	0%	0.00007	0%	0.00007	0%
Prochloraz	13	0.00136	5%	0.00118	4%	0.00300	11%	0.00869	33%
Procymidone	17	0.00225	14%	0.00178	11%	0.00744	47%	0.01199	75%
Profenofos	1	0.00004	-	0.00004	-	0.00004	-	0.00004	-
Propamocarb	13	0.00219	1%	0.00177	0%	0.00553	1%	0.01454	4%
Propiconazole	4	0.00009	0%	0.00010	0%	0.00015	0%	0.00018	0%
Pymetrozine	9	0.00021	1%	0.00021	1%	0.00041	1%	0.00080	2%
Pyraclostrobin	10	0.00168	8%	0.00197	10%	0.00576	29%	0.00633	32%
Pyridaben	6	0.00186	28%	0.00195	29%	0.00524 *	79%	0.00799 *	120%
Pyridalyl	13	0.00050	2%	0.00049	2%	0.00116	4%	0.00178	7%
Pyrimethanil	5	0.00027	0%	0.00031	0%	0.00061	0%	0.00081	1%
Pyriproxyfen	1	0.00004	0%	0.00004	0%	0.00004	0%	0.00004	0%
Simazine	1	0.00006	-	0.00006	-	0.00006	-	0.00006	-
Spinetoram	5	0.00005	1%	0.00006	1%	0.00006	1%	0.00007	1%
Spinosad	7	0.00042	3%	0.00018	1%	0.00109	7%	0.00239	15%
Spirodiclofen	1	0.00570	48%	0.00570	48%	0.00570	48%	0.00570	48%
Spiromesifen	1	0.00007	0%	0.00007	0%	0.00007	0%	0.00007	0%
Spirotetramat	6	0.00020	0%	0.00030	0%	0.00037	1%	0.00040	1%
Spiroxamine	16	0.00103	5%	0.00179	9%	0.00232	12%	0.00294	15%
Tebuconazole	8	0.00092	2%	0.00092	2%	0.00247	6%	0.00426	11%
Tebufenozide	1	0.00044	4%	0.00044	4%	0.00044	4%	0.00044	4%
Tebufenpyrad	2	0.00046	3%	0.00066	5%	0.00077	6%	0.00085	6%
Tetraconazole	1	0.00011	0%	0.00011	0%	0.00011	0%	0.00011	0%
Tetrahydrophtalimide	1	0.00006	-	0.00006	-	0.00006	-	0.00006	-
Tetramethrine	1	0.00012	0%	0.00014	0%	0.00015	0%	0.00016	0%
Thiabendazole	2	0.00099	4%	0.00065	2%	0.00292	11%	0.00506	19%
Thiacloprid	9	0.00084	1%	0.00065	1%	0.00252	2%	0.00319	3%
Thiametoxam	11	0.00025	0%	0.00037	0%	0.00055	1%	0.00066	1%
Thiophanate methyl	7	0.00010	0%	0.00012	0%	0.00015	0%	0.00016	0%
Tolclofos-methyl	4	0.00015	0%	0.00015	0%	0.00015	0%	0.00015	0%
Triadimefon (triadimenol)	1	0.00046	1%	0.00067	1%	0.00106	1%	0.00151	2%
Trifloxystrobin	10	0.00008	0%	0.00008	0%	0.00008	0%	0.00008	0%
Triflumizole	1	0.00016	0%	0.00021	1%	0.00025	1%	0.00027	1%

## References

[1] Cooper J., Dobson H. (2007). The benefits of pesticides to mankind and the environment. Crop Prot..

[2] Bethke J.A., Cloyd R.A. (2009). Pesticide use in ornamental production: What are the benefits?. Pest Manag. Sci..

[3] Morse D.L., Baker E.L., Landrigan P.J. (1979). Cut flowers: A potential pesticide hazard. Am. J. Public Health.

[4] Toumi K., Vleminckx C., Van Loco J., Schiffers B. (2016). Pesticide residues on three cut flower species and potential exposure of florists in Belgium. Int. J. Environ. Res. Public Health.

[5] Toumi K., Vleminckx C., Van Loco J., Schiffers B. (2016). A survey of pesticides residues in cut flowers from various countries. Commun. Appl. Biol. Sci. Ghent Univ..

[6] Rikken M. Le Marché Européen des Fleurs et Plantes Équitables et Durables (The European Market for Equitable and Sustainable Flowers and Plants). http://www.befair.be/sites/default/files/all-files/brochure/Le%20march%C3%A9%20europ%C3%A9en%20des%20fleurs%20et%20plantes%20%C3%A9quitables%20et%20dur%E2%80%A6_0.pdf.

[7] Baldi I., Cordier S., Coumoul X., Elbaz A., Gamet-Payrastre L., Le Bailly P. (2013). Pesticides: Effets sur la Santé.

[8] World Health Organization (2004). The WHO Recommended Classification of Pesticides by Hazard and Guidelines to Classification 2004.

[9] Dasgupta S., Meisner C. (2005). Health Effects and Pesticide Perception as Determinants of Pesticide Use: Evidence from Bangladesh.

[10] Fleming L.E., Bean J.A., Rudolph M., Hamilton K. (1999). Cancer incidence in a cohort of licensed pesticide applicators in Florida. J. Occup. Environ. Med..

[11] Munnia A., Puntoni R., Merlo F., Parodi S., Peluso M. (1999). Exposure to agrochemicals and DNA adducts in Western Liguria, Italy. Environ. Mol. Mutagen..

[12] Bolognesi C. (2003). Genotoxicity of pesticides: A review of human biomonitoring studies. Mutat. Res..

[13] Restrepo M., Munoz N., Day N.E., Parra J.E., de Romero L., Nguyen-Dinh X. (1990). Prevalence of adverse reproductive outcomes in a population occupationally exposed to pesticides in Colombia. Scand. J. Work Environ. Health.

[14] Lu J.L. (2005). Risk factors to pesticide exposure and associated health symptoms among cut-flower farmers. Int. J. Environ. Health Res..

[15] European Food Safety Authority (EFSA) (2014). Guidance on the assessment of exposure of operators, workers, residents and bystanders in risk assessment for plant protection products. EFSA J..

[16] U.S. Environmental Protection Agency (U.S. EPA) (1986). Pesticide Assessment Guidelines, Subdivision U, Applicator Exposure Moniotoring.

[17] Brouwer D.H., Brouwer R., Mik G.D., Maas C.L., Hemmen J.J.V. (1992). Pesticides in the cultivation of carnations in greenhouses: Part I—Exposure and concomitant health risk. Am. Ind. Hyg. Assoc. J..

[18] Brouwer R., Brouwer D.H., Tijssen S.C., Hemmen J.J.V. (1992). Pesticides in the cultivation of carnations in greenhouses: Part II—Relationship between foliar residues and exposures. Am. Ind. Hyg. Assoc. J..

[19] Kangas J., Manninen A., Liesivuori J. (1995). Occupational exposure to pesticides in Finland. Int. J. Environ. Anal. Chem..

[20] Methner M.M., Fenske R.A. (1996). Pesticide exposure during greenhouse applications. III. Variable exposure due to ventilation conditions and spray pressure. Appl. Occup. Environ. Hyg..

[21] Illing H.P.A. (1997). Is working in greenhouses healthy? Evidence concerning the toxic risks that might affect greenhouse workers. Occup. Med..

[22] Ecobichon D.J. (1998). Occupational Hazards of Pesticide Exposure: Sampling, Monitoring, Measuring.

[23] Van Hemmen J.J., Brouwer D.H. (1995). Assessment of dermal exposure to chemicals. Sci. Total Environ..

[24] Rajan-Sithamparanadarajah R., Roff M., Delgado P., Eriksson K., Fransman W., Gijsbers J.H.J., Van Hemmen J.J. (2004). Patterns of dermal exposure to hazardous substances in European Union workplaces. Ann. Occup. Hyg..

[25] Lesmes-Fabian C., García-Santos G., Leuenberger F., Nuyttens D., Binder C.R. (2012). Dermal exposure assessment of pesticide use: The case of sprayers in potato farms in the Colombian highlands. Sci. Total Environ..

[26] U.S. EPA (U.S. Environmental Protection Agency) (2000). Agricultural Transfer Coefficients.

[27] U.S. EPA (U.S. Environmental Protection Agency) (2001). Science Advisory Council for Exposure, Policy Number 12, Recommended Revisions to the Standard Operating Procedures (SOPs) for Residential Exposure Assessments.

[28] EU—Pesticides Database. ec.europa.eu/food/plant/pesticides/eu-pesticides-database/public/?event=activesubstance.selection&language=EN.

[29] Brouwer R., Marquart H., de Mik G., Van Hemmen J.J. (1992). Risk assessment of dermal exposure of greenhouse workers to pesticides after re-entry. Arch. Environ. Contam. Toxicol..

[30] Organisation for Economic Co-operation and Development (1997). Guidance Document for the Conduct of Studies of Occupational Exposure to Pesticides during Agricultural Application.

[31] Association of Official Analytical Chemists (2007). AOAC official method 2007.01 pesticide residues in Foods by acetonitrile extraction and partitioning with magnesium sulfate gas chromatography/mass spectrometry and liquid chromatography/tandem mass spectrometry first action 2007. J. AOAC Int..

[32] European Commission (2015). Guidance Document on Analytical Quality Control and Method Validation Procedures for Pesticides Residues Analysis in Food and Feed.

[33] Van Loco J., Beernaert H. An alternative method validation strategy for the European Decision 2002/657/EC. Proceedings of the Euro Food Chem XII: Strategies for Safe Food.

[34] U.S. EPA (U.S. Environmental Protection Agency) (2007). Dermal Exposure Assessment: A Summary of EPA Approaches.

[35] European Food Safety Authority (EFSA) (2012). Guidance on selected default values to be used by the EFSA Scientific Committee, Scientific Panels and Units in the absence of actual measured data. EFSA J..

[36] AGP—List of Pesticides Evaluated by JMPS and JMPR. www.fao.org/agriculture/crops/thematic-sitemap/theme/pests/lpe/en/.

[37] FAO Plant Production and Protection Paper Series JMPR Reports. www.who.int/foodsafety/publications/jmpr-reports/en/.

[38] Joint Meeting on Pesticide Residues (JMPR) Monographs & Evaluations. The International Programme on Chemical Safety Website. www.inchem.org/pages/jmpr.html.

[39] European Commission. Regulation (EC) (2009). No. 1272/2008 of the European Parliament and of the Council of 16 December 2008.

[40] Popendorf W.J., Leffingwell J.T. (1982). Regulating OP pesticide residues for farmworker protection. Residue Reviews.

[41] Nigg H.N., Stamper J.H., Queen R.M. (1984). The development and use of a universal model to predict tree crop harvester pesticide exposure. Am. Ind. Hyg. Assoc. J..

[42] Zweig G., Leffingwell J.T., Popendorf W. (1985). The relationship between dermal pesticide exposure by fruit harvesters and dislodgeable foliar residues. J. Environ. Sci. Health Part B.

[43] Thongsinthusak T., Ross J., Fong H., Formoli T., Krieger R. (1990). Estimation of Exposure of Persons in California to Pesticide Products That Contain Abamectin. HS-1567.

[44] McCurdy S.A., Hansen M.E., Weisskopf C.P., Lopez R.L., Schneider F., Spencer J., Schenker M.B. (1994). Assessment of azinphos-methyl exposure in California peach harvest workers. Arch. Environ. Health Int. J..

[45] Krieger R.I., Dinoff T.M. (2000). Malathion deposition, metabolite clearance, and cholinesterase status of date dusters and harvesters in California. Arch. Environ. Contam. Toxicol..

[46] Gomes J., Lloyd O.L., Revitt D.M. (1999). The influence of personal protection, environmental hygiene and exposure to pesticides on the health of immigrant farm workers in a desert country. Int. Arch. Occup. Environ. Health.

